# The Austronesian Basic Vocabulary Database: From Bioinformatics to Lexomics

**DOI:** 10.4137/ebo.s893

**Published:** 2008-11-03

**Authors:** Simon J. Greenhill, Robert Blust, Russell D. Gray

**Affiliations:** 1 Department of Psychology, The University of Auckland, Private Bag 92019, Auckland 1142, New Zealand; 2 Department of Linguistics, University of Hawai’i at Manoa, 1890 East-West Road, Moore Hall 569, Honolulu, HI 96822

**Keywords:** austronesian, linguistics, lexicon, database, ABVD

## Abstract

Phylogenetic methods have revolutionised evolutionary biology and have recently been applied to studies of linguistic and cultural evolution. However, the basic comparative data on the languages of the world required for these analyses is often widely dispersed in hard to obtain sources. Here we outline how our Austronesian Basic Vocabulary Database (ABVD) helps remedy this situation by collating wordlists from over 500 languages into one web-accessible database. We describe the technology underlying the ABVD and discuss the benefits that an evolutionary bioinformatic approach can provide. These include facilitating computational comparative linguistic research, answering questions about human prehistory, enabling syntheses with genetic data, and safe-guarding fragile linguistic information.

## Introduction

Phylogenetic methods have revolutionised biology and currently provide the most powerful way of testing evolutionary hypotheses (Harvey and Pagel, 1991; Huelsenbeck and Rannala, 1997; Pagel, 1999). To make accurate inferences computational phylogenetic methods require large amounts of well-sampled data. In biology the growth of databases has been explosive. A recent survey lists 1,078 databases in the field of molecular biology, with 110 of these added in 2007 alone (Galperin, 2008). One of these, *GenBank*^®^ (http://www.ncbi.nlm.nih.gov), contains over 80 million DNA sequences from over 260,000 organisms and doubles in size approximately every 18 months (Benson et al. 2008). Databases like *GenBank* have become crucial to modern biological research: “Access to (databases) is as important to scientific progress today as is access to a laboratory or library” (Ellis and Attwood, 2001, p. 509). In this paper we will discuss how we have applied a similar combination of phylogenetic “tree thinking” (O’Hara, 1988) and “database thinking” to languages. We will begin with a review of work exploring the relationships between genetics and linguistics, and how computational phylogenetic methods have begun to explore questions about languages. We will then proceed to describe the Austronesian Basic Vocabulary Database and the benefits that lexical data can provide.

Research into the relationship between linguistic and genetic diversity has a long history dating back at least 40 years. For example, Howells (1966) found a correlation between morphological differences and languages on the island of Bougainville, and Spielman et al. (1974) showed a strong relationship between blood group and linguistic divergence in Yanomamö Indians in southern Venezuela. One of the most influential studies reported a worldwide correlation between trees derived from the major human blood groups and a global classification of languages (Cavalli-Sforza et al. 1988). This paper was promptly criticised on a number of methodological grounds (Bateman et al. 1990). The most telling of these criticisms was that the language classification used (Ruhlen, 1987) had been constructed using a “multilateral comparison” method that most linguists consider inherently flawed (Matisoff, 1990). At a local geographic level studies have found more convincing evidence of a linkage between genes and languages. Sokal (1988) reported stronger correlations between linguistic and genetic distance (calculated from allele frequencies), than between genetic and geographic distances in Europe. Furthermore, linguistic boundaries in Europe often show zones of sharp genetic change (Barbujani and Sokal, 1990). These results indicate that language affiliation—at least in Europe—can maintain or even cause genetic differences between human populations. Recent studies have revealed a more complex picture—while the correlation between languages and genes is strong in Europe and East and Central Asia, the relationship is much weaker in other regions such as West Africa and South-East Asia (Nettle and Harriss, 2003). Hunley et al. (2007) has shown that linguistic and genetic classifications correspond well in Native and Central America, but only for the more recent splits; the deeper branches in these classifications are incongruent. One of the most fine-grained analyses (Lansing et al. 2007) has shown a very strong correlation between languages, Y chromosome lineages, and geography on the Indonesian island of Sumba. However, with the notable exception of Lansing et al. (2007), a common problem with many of the studies discussed above is that they have used arbitrary and often subjective measures of linguistic distance. A true synthesis of our linguistic and genetic histories requires quantitative analyses of both.

Recently, computational phylogenetic methods derived from evolutionary biology have been used to answer questions about the evolution of language and culture (Mace et al. 2005; Gray et al. 2007). These studies have tested scenarios about the origin of language families such as Indo-European (Gray and Atkinson, 2003) and Bantu (Holden, 2002; Holden and Gray, 2006). Other studies have explored more general factors in language evolution, such as the suggestion that structural features may reveal historical signals in Papuan languages dating back around 10,000 years (Dunn et al. 2005), and testing if the frequency of word use predicts the rates that words change (Pagel et al. 2007). A recent study (Atkinson et al. 2008) demonstrated that languages show punctuational bursts of change much like species do (Pagel et al. 2006), consistent with the claim that speakers often use their language as a social tool for demarcating groups and increasing group cohesion. Just as biologists use molecular phylogenies to test hypotheses about character evolution, anthropologists have started to use the language phylogenies as a backbone for testing hypotheses about cultural evolution (Gray et al. 2007) such as the co-evolution between the spread of cattle and subsequent loss of matriliny (Holden and Mace, 2005). However, a major impediment to the growth of phylogenetic studies of cultural evolution is the limited number of quantitative language phylogenies available.

The Pacific has long been regarded as an ideal natural laboratory for studies of linguistic and cultural evolution. The Austronesians settled the region from Taiwan, spreading into Island South-East Asia and coastal New Guinea before reaching as far as Madagascar, Micronesia, Hawaii, Rapanui (Easter Island), and New Zealand (Bellwood et al. 1995). This represents one of the greatest human migrations of all time—a feat that is made all the more impressive because it occurred before the advent of modern sailing technology. This population expansion produced the largest language family in the world, containing approximately 1,200 Austronesian languages (Gordon, 2005). Prominent Austronesian languages today include Tagalog spoken by around 21.5 million people in the Philippines, Indonesian with 30 million first-language speakers, Javanese with 90 million speakers, and New Zealand Maori with around 130,000 speakers (Blust in press). The origin and dispersal sequence of these Austronesian-speaking people is a topic of considerable debate. Most linguists and archaeologists argue for the emergence of Austronesian in Taiwan around 5,500 years ago (e.g. Blust, 1995; Blust, 1999; Diamond and Bellwood, 2003; Pawley, 2002). According to this “Out of Taiwan” scenario the Austronesians rapidly expanded, perhaps in a series of pulses and pauses (Blust, 1999; Pawley, 2002; Green, 2003), south into Island South-East Asia and east along the north coast of New Guinea into the Pacific, before spreading to the far reaches of Polynesia. In contrast, some geneticists have argued for a deeper origin in Island South-East Asia around 13,000–17,000 years ago. In this alternative scenario there were two expansions: an expansion north into Taiwan and a move east along New Guinea and into the Pacific (Oppenheimer and Richards, 2001).

In previous studies we tested the “Out of Taiwan” scenario by conducting phylogenetic analyses of lexical data derived from Blust’s Austronesian Comparative Dictionary Project (Blust nd; Gray and Jordan, 2000; Greenhill and Gray, 2005). The resulting phylogenetic trees were consistent with the sequence predicted by the “Out of Taiwan” scenario. However, the data we used for these analyses had been collected for a large comparative dictionary rather than for computational phylogenetic analyses. These data were not therefore ideally suited to making robust inferences about the sequence and timing of population expansions. Geneticists today typically explore the genes that fit the timescale they wish to make inferences about (for example, no one would sequence the rapidly evolving mitochondrial control region to resolve the deeper branches of the tree of life). Basic vocabulary provides an ideal source of data that can take full advantage of the power of phylogenetic methods to explore human linguistic and cultural history. Basic vocabulary is ideal because it changes at a slower rate than other aspects of the lexicon (Swadesh, 1952), and is less likely to be borrowed between languages (Embleton, 1986). Unfortunately, in contrast to biology, linguistics has not undergone a database revolution. The vast majority of basic vocabulary word lists, and indeed most linguistic data, are scattered in numerous obscure sources. Often these are locked away in filing cabinets in the form of unpublished manuscripts or field notebooks. Many of the basic published resources are also very hard to find—some important dictionaries are now over 100 years old (e.g. Hardeland, 1859; Aymonier and Cabaton, 1906).

The absence of large linguistic databases has some major drawbacks. First, the information is difficult to obtain; one either needs access to a world class library, or a world class linguist. Second, even if this information can be obtained it is often not documented in a consistent fashion. There is no central linguistic “GenBank” where one can find information on any language coded in a consistent manner. This scattering of information and absence of consistent coding makes large-scale comparative work extremely difficult. Third, existing linguistic databases are unsuitable for large-scale comparative analyses. The best exemplar is the Summer Institute of Linguistics’ *Ethnologue* (Gordon, 2005, http://www.ethnologue.com), which despite being an excellent linguistic resource, is primarily a worldwide Bible translation project (Erard, 2005). The other existing databases are either focused on a single language (e.g. *WordNet*, http://wordnet.princeton.edu), or contain no information about the provenance or quality of the linguistic information (e.g. *The Rosetta Project*, http://rosettaproject.org/), or are private hobby databases with limited public accessibility (e.g. *STEDT*, http://stedt.berkeley.edu/), or are inextricably linked to dubious language subgrouping proposals (e.g. *StarLing*, http://starling.rinet.ru). Finally, much of the basic information on the languages of the world is unfortunately quite fragile. Substantial amounts of comparative linguistic data can only be found in disintegrating field notes and recordings. Sometimes this information may be the only evidence that a language—and a culture—ever existed. The projects to properly store this information, like *PARADISEC* (http://paradisec.org.au/), are seriously under-funded. The fragility of current linguistic data storage is made all the more worrying by the fact that, on average, a language goes extinct every two weeks. At least a half of the world’s languages are expected to go extinct in the next century (Nettle and Romaine, 2000).

As a partial solution to the absence of consistently coded comparative lexical data, one of us (Gray) approached Robert Blust at the University of Hawaii with the idea of producing an electronic database of Blust’s extensive collection of Austronesian wordlists. Blust had collected basic vocabulary wordlists from a total of 231 Austronesian languages in order to test variation in retention rates as part of a general critique of lexicostatistical methods of language subgrouping (Blust, 1981, 2000). These wordlists contained 200 items of basic vocabulary such as words for body parts, kinship terms, simple verbs, and colours (Table 1). As we noted earlier, basic vocabulary is thought to be both relatively resistant to borrowing and more stable than other parts of the lexicon (Swadesh, 1952).

We took the 231 word-lists collected by Blust, expanded them from 200 to 210 items of basic vocabulary (Table 1), and entered them into a relational database. This was subsequently placed on the internet as the *Austronesian Basic Vocabulary Database* (“ABVD”, http://language.psy.auckland.ac.nz), where it has grown substantially. In the following sections we will describe the database structure, detail some of the features of the ABVD web application, and describe its usage. We finish by outlining how evolutionary bioinformatics ideas could be extended to create a new field of study—a field that could be dubbed, with a hint of a grin, “lexomics”.

## Database Structure and Data Content

**Figure 1:**
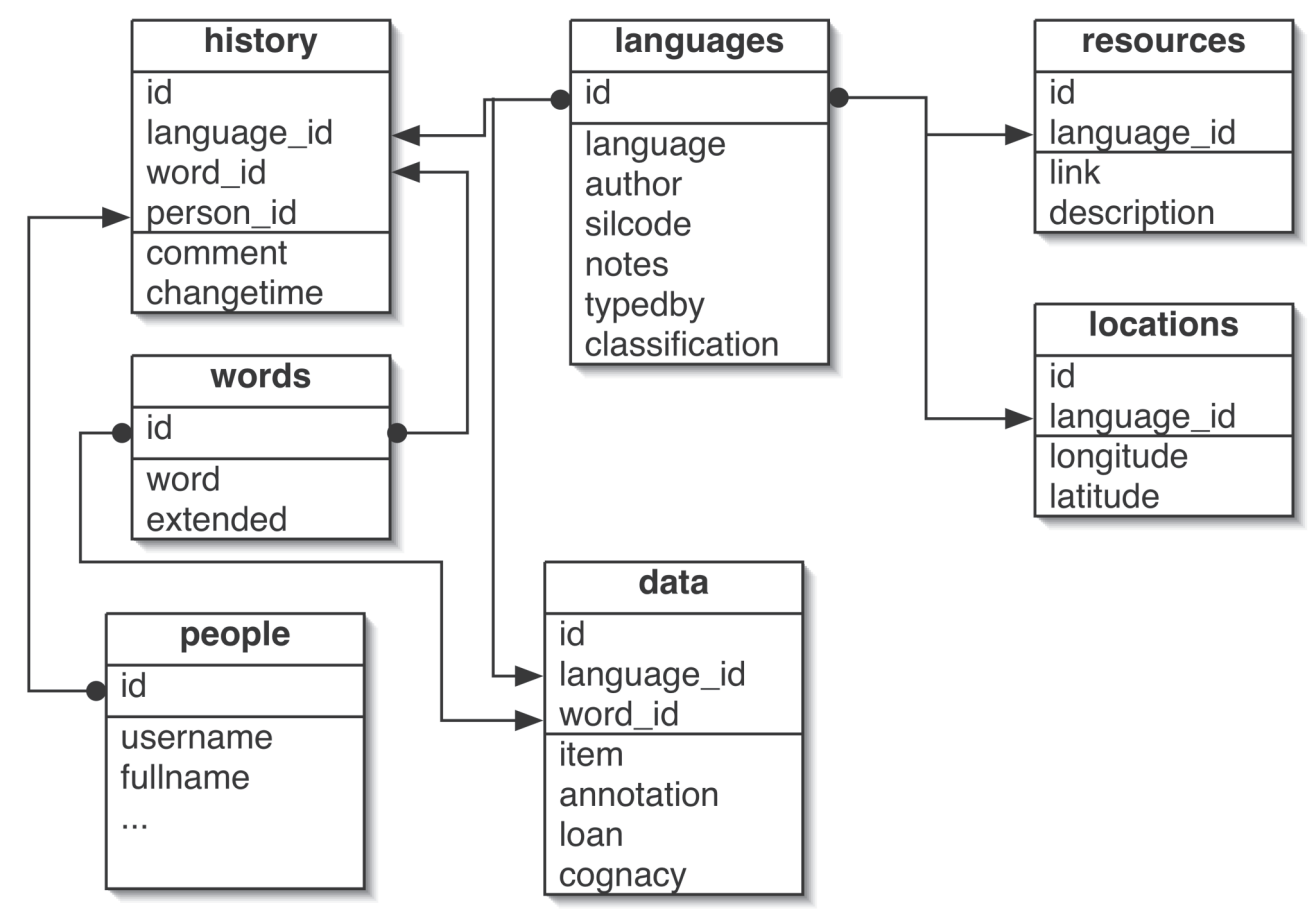
Core database schema of the Austronesian Basic Vocabulary Database.

The lexical and cognate data in the ABVD is stored in the open-source relational database *MySQL* as a series of database tables linked by standard foreign key architecture. Due to the extended and phonetic characters used for the lexical orthography, all information is encoded in the Unicode format UTF-8. The core database schema of the ABVD is shown in Figure 1. The table *languages* stores information about each language. This includes the name of language (“language”), the data source information (“author”), and the name of the person entering the data (“typedby”). The field “silcode” contains the ISO 639-3 language identification code. This identification code provides a way of linking each language to broader information about the language at other resources such as SIL International’s *Ethnologue* (http://www.ethnologue.com, Gordon, 2005), or the *World Atlas of Language Structures* (http://www.wals.info, Haspelmath et al. 2005). The “classification” field stores the current classification of the language, obtained from the *Ethnologue* classification. Finally, there is a text field (“notes”) for any extra information about the language. The *languages* table is linked to the *locations* table via a one-to-many foreign key. The *locations* table stores geographical information (“latitude”, “longitude”) about each language to enable plotting of the languages on a map. The *resources* table is also linked to the *languages* table via foreign key. This table contains links to other relevant websites about a language, such as *Wikipedia* entries, homepages of research groups investigating the language, or other online resources like dictionaries.

The table “words” contains information about the word meaning categories in the database. Each word category (Table 1) has an entry with a short form entered in the “word” field, and a descriptive form in the “extended” field where necessary. For example, word #13 “back” has extended information (“body part”) to clarify that this category relates to the body part, and not the direction.

The table *data* contains the lexical entries and is the main data store for the database. Each entry in this table is linked to a language in *languages* and to a word in *words* via foreign key. This allows each language to have multiple entries for each word. For example, Nukuoro has three entries in the word meaning category for “hair”: “ngangailu” (hair on head), “ngae” (a single hair on the head), and “hulu” (body hair). The field “item” contains the lexical entry itself. The “annotations” field contains any comments about this item, such as slightly different meanings, or information about irregular sound change. If the entry is known to be borrowed from another language, the field “loan” is used to flag this status. The “cognacy” field contains information about cognate set membership for this entry, that links words that have evolved from a common ancestor (see below).

All changes to the data are tracked using the *history* table. The information logged for each change includes which language and word were modified (“language_id”, “word_id”). This is supplemented with a comment field (“comment”) for annotating the change, and a timestamp of when the change occurred (“changetime”). The field “person_id” denotes the editor who made the change, and is a foreign key onto the table *people* that stores user information, access credentials, etc.

## Data Sources

The data in the ABVD comes from three primary sources. The first source of data are wordlists collected by linguists during fieldwork. The major providers have been Robert Blust, John Lynch, and Malcolm Ross. Many other linguists have graciously contributed word lists for languages they are familiar with. The second primary source of data has been published wordlists and dictionaries. The major publications mined for information were the Polynesian Lexicon project POLLEX (Biggs and Clark, 2000), and a large collection of Micronesian reconstructions (Bender et al. 2003a, 2003b). This was augmented with a number of publications describing languages from Taiwan (Ferrell, 1969, 1982), the Batanes Islands (Tsuchida et al. 1987), the Moluccas (Taber, 1993), the Solomons (Tryon and Hackman, 1983), Vanuatu (e.g. Crowley, 2006a, 2006b, 2006c), and the Philippines (Reid, 1971), as well as many others. The final primary source of data in the ABVD has come from native speakers who have contributed word lists for their languages through the web interface (see: http://language.psy.auckland.ac.nz/austronesian/people.php#authors for a full contributor list). As the ABVD has grown, a number of languages from outside the family have been incorporated for comparative purposes. These languages include the Sino-Tibetan language Old Chinese and the Tai-Kadai language Buyang (both added by Laurent Sagart).

## Cognate Judgements

Just as biologists are interested in homologous genes to trace ancestry, linguists are interested in homologous words. These homologous words—cognates—can be identified using systematic sound correspondences between words of similar meaning across languages. For example, Table 2 shows a number of word forms in five Polynesian languages with the cognate words color-coded. In the entries for “hand”, the forms show a common “l” to “r” sound shift. This is also seen in the entries for “skin”, with a systematic correspondence between Hawaiian’s “l” and Tahitian/Maori/Rapanui’s “r”. Another systematic correspondence can be seen in the entries for “bone” and “woman”. These correspondences can be used to identify the words (and hence the languages) that have descended from a common ancestor. In this case, the forms colored in light blue share a common ancestor. In the entries for “to spit”, there are two cognate sets—the first “anu/aanu” is present in Samoan and Rapanui and descends from the ancestral Nuclear Polynesian form *anu, whilst the second “tuhu/tutuha” is an innovation in the East Polynesian languages of Tahitian and Maori. This cognate set information can be easily encoded in a binary matrix reflecting the presence or absence of cognates. Such a matrix is well suited to analyses using phylogenetic methods (e.g. Gray and Jordan, 2000; Gray and Atkinson, 2003).

Cognate judgements were done by or in consultation with a number of linguistic experts. Robert Blust provided cognate decisions for most areas within the Austronesian family. Jeff Marck assessed the languages of Polynesia, Micronesia and neighbouring regions for cognation. John Lynch assessed Vanuatu and New Caledonia languages, and Malcolm Ross assisted with the cognation judgements of languages in Near Oceania. Laurent Sagart provided cognation judgements for a number of Formosan languages. Graham Thurgood provided the judgements for the Chamic language subgroup. Russell Gray, Simon Greenhill and Cordelia Nickelsen assessed the remaining regions in consultation with Robert Blust. Since these cognate decisions generally require a high level of linguistic expertise, only the database administrators can edit the cognate coding.

## User Interface

The ABVD web interface is implemented in the programming language PHP running on an Apache webserver. The interface has a number of core functions: displaying information about languages, displaying information about words, enabling new data to be entered, searching through the data, and allowing editors to maintain the database. These functions will be discussed in turn.

### Languages page

**Figure 2:**
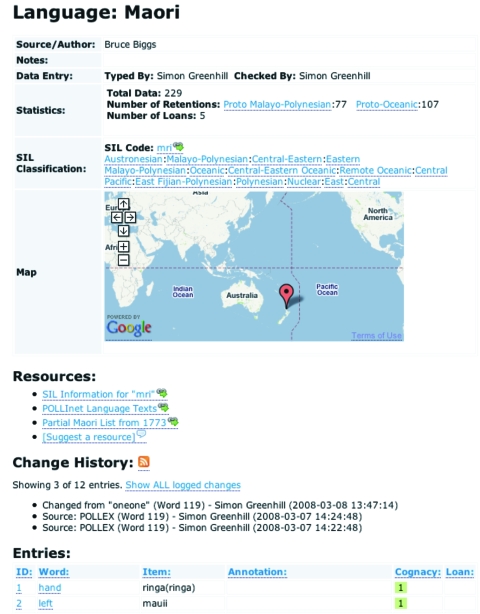
The languages page showing information for the language Maori.

The *languages* page displays all the information about a given language (Fig. 2). First, the available information about the language is displayed, including the language name, the data source, notes about the language, and links to the *Ethnologue* page for more information. The geographical location of the language is displayed using the *Google Maps* web service (http://maps.google.com). This is followed by links to external resources relevant to this language from the *resources* table (e.g. the Maori list links to an alternate wordlist from 1773, and to a language text in POLLInet http://bilbo.ling.su.se/pollinet/). Finally, the wordlist for this language is displayed, showing the lexical entries, annotations and judgements of cognation. All changes to this language are also published via RSS 2.0 feeds, allowing users to subscribe and be notified of any changes. To enable the data to be used “offline”, users can download the information for a language in comma-separated or XML format.

### Words page

**Figure 3:**
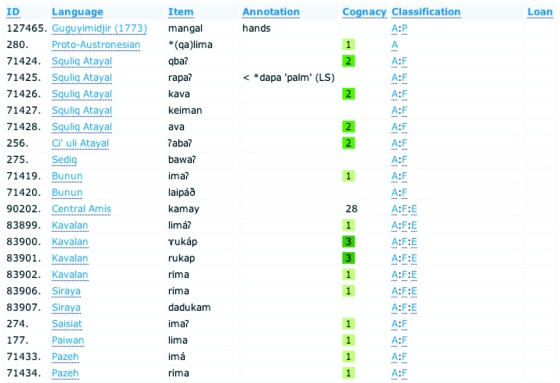
The words page showing entries for the word meaning category “hand”.

The user is able to display all the entries for a given word meaning category using the *words* page (Fig. 3). For example, if word #1 “hand” is selected, the user will see all entries in all languages that mean “hand”, followed by any annotations, the cognate information, an abbreviated language classification, and a flag denoting loan status. This interface enables the sorting of information by any of the fields, including grouping alphabetically by lexical item, by language classification, and by grouping all the items from each cognate set together.

### Data entry

A large proportion of the data in the ABVD has been added by visitors to the website. There are two ways to add new information to the database. Every language page has a link to a comment form where users can leave annotations on entries, suggest new entries, or correct existing entries. Additionally, for more large-scale data entry needs, the user can use the *webedit* interface that facilitates the entering of new data for new languages. This webedit page can be transformed to match a number of common wordlist formats. To assist the user in entering phonetic characters, we have implemented a character “chooser” in javascript. This enables these extended characters to be easily inserted into records.

### Search

A key function for the ABVD is the ability to search through the entries. To this end, a search interface was implemented, allowing users to search for languages, word meaning categories, authors, or within the lexical entries.

### Editors section

The Editors section of the website provides a number of editorial functions for ABVD curators. First, it tracks and stores all incoming data to the website. This can then be checked, before being added to the main database for public display. Second, the editors section implements editing functions for all of the data in the database. Third, the editors’ section also contains a specialised interface to facilitate cognate judgments.

## Statistics and Usage

**Figure 4:**
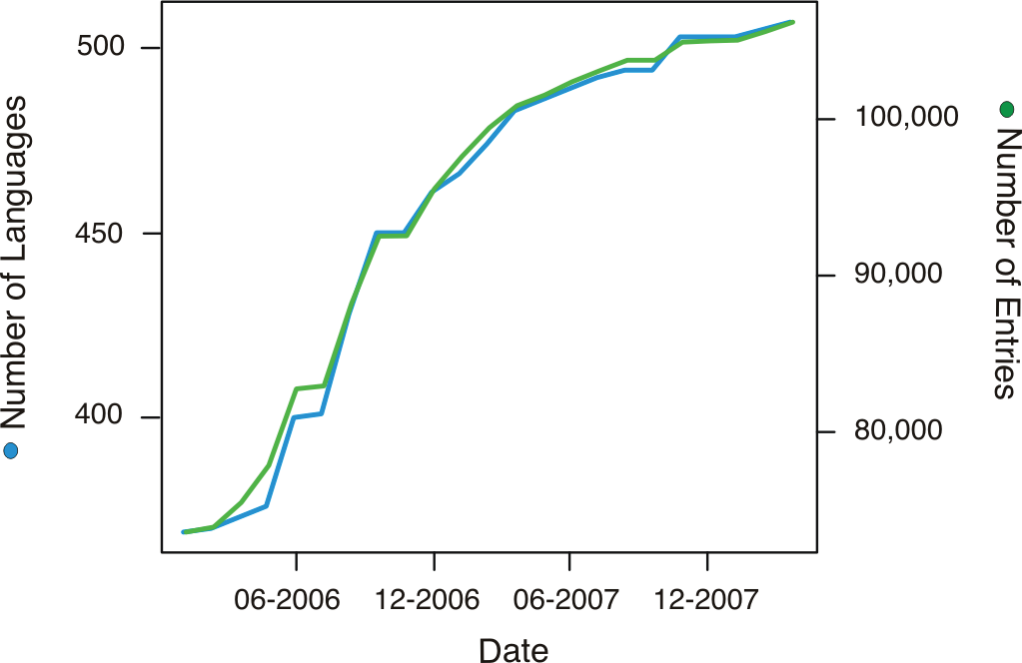
The growth of the Austronesian Basic Vocabulary Database over the last two years (01–01–2006 – 01–04–2008).

**Figure 5:**
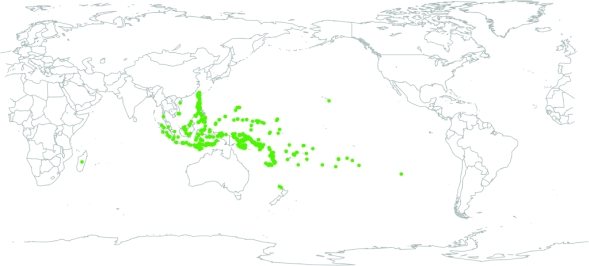
Map showing the approximate location of the languages currently attested in the Austronesian Basic Vocabulary Database.

Currently the ABVD has grown from the original set of 231 languages to over 500 languages in the Pacific region (Fig. 4). This represents a sample from around half of the 1,200 languages in the Austronesian language family. It contains a good coverage of Austronesian language subgroups from across the Austronesian-speaking region (Fig. 5). In total, there are more than 100,000 lexical entries in the ABVD. Most languages are well-attested, with an average of 209 entries per language. Some languages are very well-attested, such as Kavalan (Taiwan), which has 456 entries. Other languages are more poorly attested due to lack of data or language extinction. The language with the fewest entries is a partial wordlist of Maori collected during Cook’s first voyage to the Pacific in 1773 (Parkinson, 1773).

The ABVD website served 1.8 million pages in the last 12 months to around 37,000 visitors, with a median of 9.5 pages per visitor. The main source of these visitors is from search engine queries about specific languages with languages like Niue, Madak, and Buginese being among the most sought-after. These users are primarily from the Asia-Pacific region, but the site has attracted users world-wide. It is currently linked to by numerous Wikipedia pages, and many other prominent websites such as the British Museum. The data in the ABVD has been used by the linguistic community in a number of publications (e.g. Atkinson et al. 2008; Blevins, 2007; Jones, 2007; McMahon and McMahon, 2008; Neeleman and Szendri, 2007).

## Recent and Future Directions

We are currently focusing on a number of enhancements to the ABVD. The first enhancement underway is the standardisation of the occasionally idiosyncratic orthography where different sources have used different symbols for the same phonemes. This is often trivial, with the velar nasal phoneme “η” sometimes coded as “ng”. However, other instances may hinder the interpretation of the lexical forms, such as where glottal stops are denoted with an apostrophe or question mark, when they are more clearly represented with the standard symbol “?”. Some users have reported problems due to the lack of standard support for UTF-8 in some older web-browsers. In these browsers, certain extended characters are replaced with a “missing” character glyph. These issues can be worked around by sending the affected characters as inline images, or by translating them on-the-fly into alternate encoding schemes like X-SAMPA (Wells, 1997), or by convincing users to upgrade to better web browsers. We favour the later option.

The second planned enhancement is the continued growth of the ABVD. We have information from at least another 200 Austronesian languages to enter, and there is much more data in the primary literature available for mining. This will be further augmented with wordlists from languages neighbouring the Austronesian language family, including those from Mon-Khmer, Tai-Kadai, Hmong-Mien, and Sino-Tibetan languages. Ultimately, we would like to construct a global database of basic information about the world’s languages. Naturally, trying to understand the histories of languages and their speakers based on a short sample of vocabulary has its limitations. For more detailed inferences this global database would ideally be extended to include additional data on morphology, phonology, structure, grammar, and typology. There has been a recent push towards large-scale genetic database projects like National Geographic and IBM’s Genographic Project (e.g. Behar et al. 2007) that aims to “map humanity’s genetic journey through the ages” (http://www.nationalgeographic.com/genographic). We see the ABVD as a first step towards a similar “Linguagraphic” Project that would aim to map humanity’s linguistic and cultural journey through the ages.

To facilitate the creation of a global database we need to abstract out common components of the databases to enable the storage of lexical information from different language families. We currently have databases under development for languages of the Mayan, Uto-Aztecan and Bantu language families. Each of these families requires slightly different information to be stored, and manipulated in different ways. For example, the Bantu languages require the lexical item and the nominal prefix to be separated. By taking the knowledge and experience we have acquired through developing the ABVD, we hope to be able to make a fully extensible database for storing a wide range of global linguistic information in future. Ideally the data in this global database would be coded in a way that facilitates analyses using computational phylogenetic methods.

## Conclusion

The Austronesian Basic Vocabulary Database provides a comprehensive comparative source of lexical data for a large number of Pacific languages. This lexical information is not only of enormous value in its own right, it also has much to offer geneticists interested in elucidating human history. Linguistic analyses can assist genetic studies by improving sampling designs. It is not uncommon for genetic studies to sample DNA from culturally meaningless groups like “Melanesians” or “Australians” (Green, 1991). Unfortunately, these “culturally challenged” analyses are difficult to integrate into the bigger picture of human prehistory. Languages are strong markers of cultural groups and their affinities (Mace and Pagel, 1994). Therefore, genetic sampling that takes linguistic affinities into account can be linked much more directly into the inferences from anthropology and archaeology.

Languages are, as the poet Ralph Waldo Emerson noted, the “archives of history” (Emerson, 1983, p. 417). The second way that lexical data can supplement genetic studies is by identifying population processes that can affect the inferences drawn from genetics. For example, the speakers of the Taiwanese language Thao have borrowed a substantial amount of lexicon from Bunun (Blust, 1996). The lexicon borrowed is largely related to words for women and other traditional female roles (e.g. cooking and child-rearing). This is probably an outcome of Thao men marrying Bunun women, and thus acquiring the vocabulary for this specific semantic domain. In this case, the use of Y chromosomal or mtDNA data would give two strikingly different accounts. Indeed, it has been suggested that the predominance of matrilineal descent patterns underlies the apparent conflict between mtDNA and Y chromosome histories in the Pacific (Hage and Marck, 2003). Lexical information can be used to identify these problematic processes and explain these sex-specific differences in admixture.

The final way that lexical data can enhance inferences about human history is through their ability to resolve relatively recent events. Our lexicon is large and rapidly evolving. In contrast, there is little information about recent human history (i.e. over the Holocene) stored in DNA. Despite mitochondrial DNA having higher mutation rates than nuclear DNA, there is still only around 1.7 × 10^−8^ substitutions per site per year across the ~16,000 base pairs (Ingman et al. 2000). Many inferences about Pacific prehistory have been drawn from the presence of a “Polynesian motif ” in mtDNA—just three substitutions in the HVR-1 region (16217C, 16247G, and 16261T, see Melton et al. 1995, Redd et al. 1995). In contrast, the dataset we are currently analysing from 400 languages extracted from the ABVD has over 34,000 characters—twice the size of the mitochondrial genome—and over 6,000 of these characters are parsimony-informative. The amount of signal in this lexical data therefore provides analyses with far greater resolution and power.

The combination of large comparative linguistic databases and computational phylogenetic methods we have advocated in this paper is the direct extension of evolutionary bioinformatic thinking to historical linguistics. This relatively new area of study could be christened “lexomics”. This nascent approach could provide a very powerful way of “triangulating” (sensu Kirch and Green, 2001) the history of cultures through the linking of genetic, linguistic, and archaeological data in a quantitative computational framework.

## Figures and Tables

**Table 1:** The 210 word meaning categories collected in the Austronesian Basic Vocabulary Database.

Adjectives	bad/evil, big, cold, correct/true, dirty, dry, dull/blunt, good, heavy, long, narrow, new, old, painful/sick, rotten, sharp, short, shy/ashamed, small, thick, thin, warm, wet, wide
Animals	bird, dog, egg, fish, louse, mosquito, rat, snake, spider, worm (earthworm)
Body Parts	back, belly, blood, bone, breast, ear, eye, feather, hair, hand, head, intestines, leg/foot, liver, mouth, neck, nose, shoulder, skin, tail, tongue, tooth, wing
Colors	black, green, red, white, yellow
Directions	above, at, below, far, in/inside, left, near, right
Numbers	one, two, three, four, five, six, seven, eight, nine, ten, twenty, fifty, one hundred, one thousand
People	child, father, he/she, husband, I, man/male, mother, name, person/human being, they, thou, we, wife, woman/female, you
Plants	branch, flower, fruit, grass, leaf, root
Other	all, and, how?, if, no/not, other, that, this, what?, when?, where?, who?
Other Nouns	ash, cloud, day, dust, earth/soil, fat/grease, fire, fog, house, lake, lightning, meat/flesh, moon, needle, night, rain, road/path, rope, salt, sand, sea, sky, smoke, star, stick/wood, stone, thatch/roof, thunder, water, wind, woods/forest, year
Verbs	to bite, to blow, to breathe, to burn, to buy, to chew, to choose, to climb, to come, to cook, to count, to cry, to cut/hack, to die/be dead, to dig, to dream, to drink, to eat, to fall, to fear, to flow, to fly, to grow, to hear, to hide, to hit, to hold, to hunt, to kill, to know/be knowledgeable, to laugh, to lie down, to live/be alive, to open/uncover, to plant, to pound/beat, to say, to scratch, to see, to sew, to shoot, to sit, to sleep, to sniff/smell, to spit, to split, to squeeze, to stab/pierce, to stand, to steal, to suck, to swell, to swim, to think, to throw, to tie up/fasten, to turn, to vomit, to walk, to work, to yawn

**Table 2:** Words meaning “hand”, “skin”, “bone”, “woman”, and “to spit” in five Polynesian languages. Cognate sets are color-coded.

Language	“hand”	“skin”	“bone”	“woman”	“to spit”
Samoan	lima	pa’u	ivi	fafine	anu
Hawaiian	lima	‘ili	iwi	wahine	pupuhi
Tahitian	rima	‘iri	ivi	vahine	tutuha
Maori	ringa(ringa)	kiri	iwi	wahine	tuha
Rapanui	rima	kiri	ivi	bahine	aanu
